# Transactions of the Ohio State Medical Society, 1862

**Published:** 1863

**Authors:** 


TRANSACTIONS OF THE OHIO STATE MEDICAL SOCIETY, 1862.

This is a neatly bound volume of 120 pages, containing the record of proceedings and papers of the Seventeenth Annual Meeting of the Ohio State Medical Society, held ot Ohio White Sulphur Springs, June 17th and 18th, 1862. After the brief record of proceedings, the next twenty pages are occupied by the Annual Address of Dr. M. B. WRIGHT, the retiring president. His subject is "The Idolatry of our People, or the Rebellion in its Medical Aspects;" and it is discussed with all the ability of its well-known author. The next paper is a Prize Essay on the Use of Anesthetics in Obstetrics, by H. CULBERTSON, M.D., of Zanesville, Ohio. It is a well-written essay, occupying about fifty pages, and is worthy of the attention of the profession. Two brief reports, one on Medical Literature and the other on the Obituary Record of the Society for the year, complete the volume.

